# Lemon Myrtle (*Backhousia citriodora*) Extract and Its Active Compound, Casuarinin, Activate Skeletal Muscle Satellite Cells In Vitro and In Vivo

**DOI:** 10.3390/nu14051078

**Published:** 2022-03-04

**Authors:** Ayumi Yamamoto, Shinichi Honda, Mineko Ogura, Masanori Kato, Ryuichi Tanigawa, Hidemi Fujino, Seiji Kawamoto

**Affiliations:** 1 Biotechnology Research Laboratories, Pharma & Supplemental Nutrition Solutions Vehicle, Kaneka Corporation, 1-8 Miyamae-cho, Takasago-cho, Takasago 676-8688, Japan; shinichi.honda@kaneka.co.jp (S.H.); mineko.tsuruoka@kaneka.co.jp (M.O.); masanori.kato@kaneka.co.jp (M.K.); 2 Unit of Biotechnology, Hiroshima Research Center for Healthy Aging (HiHA), Graduate School of Integrated Sciences for Life, Hiroshima University, 1-3-1 Kagamiyama, Higashi-Hiroshima 739-8530, Japan; 3 Analysis Division, Osaka Analysis Center, Kaneka Techno Research Corporation, 5-1-1 Torikainishi, Settsu, Osaka 566-0072, Japan; ryuichi.tanigawa@kaneka.co.jp; 4 Department of Rehabilitation Science, Graduate School of Health Sciences, Kobe University, 7-10-2 Tomogaoka, Suma-ku, Kobe 654-0142, Japan; fujino@phoenix.kobe-u.ac.jp

**Keywords:** sarcopenia, skeletal muscle satellite cells, lemon myrtle, *Backhousia citriodora*, casuarinin, ellagitannin, interleukin-6

## Abstract

Sarcopenia is an age-related skeletal muscle atrophy. Exercise is effective in improving sarcopenia via two mechanisms: activation of skeletal muscle satellite cells (SCs) and stimulation of muscle protein synthesis. In contrast, most nutritional approaches for improving sarcopenia focus mainly on muscle protein synthesis, and little is known about SC activation. Here, we investigated the effect of lemon myrtle extract (LM) on SC activation both in vitro and in vivo. Primary SCs or myoblast cell lines were treated with LM or its derived compounds, and incorporation of 5-bromo-2′-deoxyuridine, an indicator of cell cycle progression, was detected by immunocytochemistry. We found that LM significantly activated SCs (*p* < 0.05), but not myoblasts. We also identified casuarinin, an ellagitannin, as the active compound in LM involved in SC activation. The structure–activity relationship analysis showed that rather than the structure of each functional group of casuarinin, its overall structure is crucial for SC activation. Furthermore, SC activation by LM and casuarinin was associated with upregulation of interleukin-6 mRNA expression, which is essential for SC activation and proliferation. Finally, oral administration of LM or casuarinin to rats showed significant activation of SCs in skeletal muscle (*p* < 0.05), suggesting that LM and casuarinin may serve as novel nutritional interventions for improving sarcopenia through activating SCs.

## 1. Introduction

Sarcopenia is an age-related skeletal muscle atrophy and is defined as a progressive and generalized skeletal muscle disorder that involves accelerated loss of muscle mass and function [1]. Progression of sarcopenia is associated with many health risks, such as an increase in falls and fractures, loss of activities of daily living, and poor quality of life [2]. Sarcopenia was estimated to affect approximately 50 million people in 2010 and this number is expected to increase as the number of older adults increases [3]. The primary intervention for improving sarcopenia is exercise, which has been shown to benefit older adults with sarcopenia [1]. Among exercise methods, resistance exercise is recommended for improving sarcopenia, but it should be performed considering the risks of a temporary increase in blood pressure [4] and injury [5]. In contrast, nutritional interventions are simple and safe approaches. Several nutritional approaches for sarcopenia have been reported, including adequate intake of protein [6], vitamin D [7], omega-3 polyunsaturated fatty acids [8], and leucine [9]. However, effective nutritional interventions for sarcopenia have not yet been established [1], partly because the differences in skeletal muscle hypertrophy mechanisms between exercise and nutritional interventions are not fully understood. 

Skeletal muscle is composed of multinucleated cells called myofibers. Since myofiber nuclei, or myonuclei, are post-mitotic and cannot divide to produce new myonuclei, new myonuclei are supplied through the proliferation of skeletal muscle satellite cells (SCs) [10]. SCs are localized between the basal membrane and the plasma membrane of myofibers [11]. In adult muscle, SCs normally reside in a quiescent and undifferentiated state. However, when skeletal muscle is stimulated by injury or exercise, SCs are activated, enter the cell cycle, and divide to produce myoblasts. Myoblasts further proliferate, differentiate, fuse into myofibers, and form new myonuclei [12]. In addition, it is known that some myoblasts return to a quiescent state to maintain the SC pool [13]. Verdijk et al. have shown that age-related skeletal muscle atrophy is accompanied by a decline in the number of SCs and that resistance exercise increases both myofiber size and SC numbers in older adults [14]. Furthermore, it has been reported in mice that the number of SCs decreases in disuse muscle atrophy but increases in stand-up exercise training after atrophy [15].

Exercise induces skeletal muscle hypertrophy through both activation of SCs and stimulation of muscle protein synthesis [16]. In contrast, most nutritional approaches induce muscle protein synthesis stimulation, but little is known about their effects on SC activation [17]. In this study, we focused on the differences in skeletal muscle hypertrophy mechanisms induced by exercise and most nutritional interventions and searched for a novel functional food ingredient that activates SCs. We found that the water extract of lemon myrtle (*Backhousia citriodora*) leaves could activate SCs in vitro; therefore, we further characterized SC activation by the lemon myrtle extract (LM) both in vitro and in vivo. Using in vitro assays, we investigated whether LM specifically activates SCs, and if so, then which compound in LM activates SCs, and by what mechanism LM activates SCs. In addition, we evaluated the effects of oral administration of LM and its active compound to rats on SC activation in skeletal muscle. 

## 2. Materials and Methods

### 2.1. Preparation of LM

Cut and dried samples of lemon myrtle leaves were supplied by Australian Native Lemon Myrtle Farms (Airlie Beach, Queensland, Australia). The weighed samples were extracted with 5-fold volume of water for 2 h at 50 °C. The extract was filtered through an α-cellulose membrane, and the supernatant was collected. These procedures were repeated twice. The supernatants from the two extractions were combined, and the solvent was evaporated under reduced pressure at 50 °C using a rotary evaporator. The concentrated extract was then freeze-dried under reduced pressure, and the lyophilized powder (LM) obtained was stored at 4 °C until further use.

### 2.2. Animals

Male Sprague-Dawley rats were purchased from Japan SLC (Shizuoka, Japan) at 13-week-old for an in vivo assay and over 6-month-old for in vitro assays. Animals were housed at constant humidity (55% ± 10%) and temperature (22 °C ± 2 °C) in a 12 h light/dark cycle and had free access to food (CE-2; Clea Japan, Tokyo, Japan) and water. Animals were acclimated to the environment for one week prior to the experiments. All experimental procedures were approved by the Animal Care and Use Committee of Kaneka Corporation (approval number: 2019–17 and 2020–6, approval date: 29 March 2019, and 31 March 2020, respectively), and conducted in accordance with the guidelines for animal experiments of Kaneka Corporation.

### 2.3. SC Isolation and Culture 

SC isolation and culture were performed according to previously described methods with slight modifications [18,19,20]. Briefly, after the rats were euthanized, the upper hindlimb and back muscles were excised, and adipose and connective tissues were trimmed. The muscle tissues were minced with scissors and digested for 1 h at 37 °C with 1.25 mg/mL protease type XIV (Sigma-Aldrich, St. Louis, MO, USA). The cells were separated from muscle fiber fragments and tissue debris by differential centrifugation and filtration through cell strainers (100 and 40 µm). Following further centrifugation, cells were suspended in one of the following media: (1) 10% HS-DMEM; Dulbecco’s modified Eagle medium (DMEM; Gibco, Grand Island, NY, USA) supplemented with 10% horse serum (HS; Gibco), 1% antibiotic-antimycotic solution (AA; Gibco), and 0.5% gentamicin (Gibco) for 5-bromo-2′-deoxyuridine (BrdU)-incorporation assay; and (2) 20% FBS-Ham’s F-10; Ham’s F-10 nutrient mixture medium (Ham’s F-10; Gibco) supplemented with 20% fetal bovine serum (FBS; Gibco), 1% AA, and 0.5% gentamicin for mRNA expression assay. For the BrdU-incorporation assay, the cells were seeded into 48-well plates coated with poly-L-lysine (Sigma-Aldrich) and fibronectin (Sigma-Aldrich) at a density of 0.5 g tissue/cm^2^. The cells were cultured for 24 h prior to the assay. For the mRNA expression assay, the cells were seeded into 90 mm dishes coated with poly-L-lysine and fibronectin at a density of 0.1 g tissue/cm^2^ and cultured for 16 h. To enrich the cell density, the cells were washed three times, harvested using 0.25% trypsin-EDTA (Gibco), and reseeded into 24-well plates coated with poly-L-lysine and fibronectin at a density of 3.5 × 10^4^ cells/well. The cells were cultured for a further 8 h prior to the assay. All cultures were maintained in a humidified atmosphere of 5% CO_2_ at 37 °C.

### 2.4. Myoblasts Culture 

L6 rat myoblast and C2C12 mouse myoblast cell lines were purchased from KAC (Kyoto, Japan) and cultured in DMEM supplemented with 10% FBS, 1% AA, and 0.5% gentamicin. For the BrdU-incorporation assay, the cells were seeded into 48-well plates at a density of 1 × 10^4^ cells/well and cultured for 24 h prior to the assay.

### 2.5. In Vitro BrdU-Incorporation Assay 

The effects of LM and its derived compounds on cell proliferation were evaluated using BrdU incorporation, which is an indicator of cell activation (entry into the cell cycle) and subsequent proliferation. Samples used for the assay are listed as follows: LM, casuarinin (isolated from LM, described in Section 2.7), gallic acid (ChromaDex, Los Angeles, CA, USA), myricitrin (Adooq Bioscience, Irvine, CA, USA), hyperin (Extrasynthese, Genay CEDEX, France), quercitrin (Extrasynthese), ellagic acid (Fujifilm Wako Pure Chemical, Osaka, Japan), casuarictin (Nagara Science, Gifu, Japan), castalagin (Sigma-Aldrich), and recombinant human hepatocyte growth factor (HGF; positive control for SC activation [21], Gibco). Each sample was dissolved in water or dimethyl sulfoxide (DMSO) and then resuspended in 10% HS-DMEM to the following final concentrations: LM (1–10 µg/mL), casuarinin (13–400 nM), gallic acid, myricitrin, hyperin, and quercitrin (25–100 nM each); ellagic acid, casuarictin, and castalagin (250 nM each); and HGF (5 ng/mL). The cells were prepared as described in Section 2.3 and Section 2.4. The BrdU-incorporation assay was performed according to previously described methods with some modifications [19,22]. Briefly, each culture was washed three times, and the cell culture medium was replaced with 10% HS-DMEM (as the control, Ctrl) or 10% HS-DMEM containing each sample described above and incubated for 22 h. At least three independent cultures were performed for each treatment. The cultures were pulse-labeled with 10 µM BrdU (Sigma-Aldrich) in 10% HS-DMEM for 2 h followed by fixation with cold methanol–H_2_O_2_ for 10 min. BrdU-positive (BrdU^+^) cells were detected by immunocytochemistry using a monoclonal anti-BrdU antibody (1:500, Sigma-Aldrich), a horseradish peroxidase (HRP)-conjugated anti-mouse IgG antibody (1:500, Exalpha Biologicals, Shirley, MA, USA), and 3,3′-diaminobenzidine tetrahydrochloride (Sigma-Aldrich). The number of cells was counted under a microscope and the ratio of BrdU^+^ cells to the total number of cells was calculated. The data are shown as fold changes compared to the Ctrl.

### 2.6. Liquid Chromatography–Tandem Mass Spectrometry (LC-MS/MS) and High-Performance Liquid Chromatography (HPLC) Analysis 

The major compounds in LM were analyzed using LC-MS/MS. A Nexera X2 UHPLC system (Shimadzu, Kyoto, Japan) equipped with a binary pump, a column oven, and a photodiode array (PDA) detector was coupled to a maXis 4G quadrupole-time-of-flight mass spectrometer equipped with an electrospray ionization (ESI) source (Bruker, Billerica, MA, USA). Chromatographic separation was performed using a YMC-Pack ODS-A column (250 × 4.6 mm, 5 µm; YMC, Kyoto, Japan). The mobile phases consisted of acetonitrile/methanol (1:1, *v*/*v*) (A) and 0.1% formic acid in water (B) in a gradient elution analysis programmed as follows: 95–85% (B) at 0–20 min, 85–70% (B) at 20–100 min, 70% (B) at 100–120 min, and 70–95% (B) at 120–140 min. The flow rate was 0.7 mL/min. The injection volume of the LM sample solution was 10 µL. The column temperature was set at 40 °C. UV spectra were recorded at 260 nm. The ESI interface was operated in the negative mode, and the mass spectra (MS) were acquired in the mass range of 50–1500 *m*/*z*. Tentative identification of compounds was performed by comparing MS and MS/MS spectra with data available on MassBank (http://www.massbank.jp/, accessed on 27 February 2022) and MetFrag (https://ipb-halle.github.io/MetFrag/, accessed on 27 February 2022) online databases.

Identification and quantification of tentatively identified peaks were performed using a prominence HPLC system (Shimadzu). Among the peaks, four peaks were identified and quantified by HPLC using analytical standards (the same materials as described in Section 2.5), whereas one peak (casuarinin) was identified by nuclear magnetic resonance (NMR) analysis (described in Section 2.7) and quantified by HPLC using chemically synthesized casuarinin [23], a kind gift from Dr. Yamada and Dr. Wakamori (Kwansei Gakuin University and Tokyo University of Agriculture, respectively). The separation conditions were the same as those described above for LC-MS/MS, except for the mobile phases, sample preparation, and sample injection volumes. The mobile phases consisted of acetonitrile/methanol (1:1, *v*/*v*) (A) and 20 mM phosphoric acid in water (B) in a gradient elution analysis programmed as follows: 95–85% (B) at 0–20 min, 85–82.2% (B) at 20–35 min, 82.2–70% (B) at 35–55 min, and 70% (B) at 55–75 min. Each sample was prepared as follows: LM was dissolved in water (2 mg/mL), and each standard was dissolved in methanol (2–200 µg/mL). Sample injection volumes were 5 µL. Peaks were detected in the range of 200–800 nm and identified by the retention time and spectrum compared with each standard. Quantification of each compound was performed using standard curves at 260 or 270 nm, and the results are shown as milligram of each compound per gram dry weight of LM.

### 2.7. Casuarinin Isolation and NMR Analysis

To determine the molecular structure of casuarinin peak estimated by LC-MS/MS, we performed the peak isolation and NMR analysis. The peak was isolated from LM using three-step column chromatography. First, LM was applied to a Diaion HP-20 column (Sigma-Aldrich), and eluted with 0%, 10%, 20%, 30%, and 40% aqueous methanol to obtain 10 fractions. Based on the HPLC chromatogram of each fraction, the fractions eluted with 20% and 30% aqueous methanol were collected. Next, the fractions were applied to a Toyopearl HW-40C column (Tosoh, Tokyo, Japan) and eluted with 50% aqueous methanol. Following fractionation, samples enriched with target peak were collected. Finally, the samples were reapplied to a Toyopearl HW-40C column using the same method as described above, and a pure target sample was isolated. The isolated sample was subsequently analyzed using an AVANCE NEO 700 NMR spectrometer (Bruker). ^1^H NMR (700 MHz) and ^13^C NMR (176 MHz) spectra were obtained in methanol-d^4^ at 30 °C using tetramethylsilane as the internal standard. The molecular structure of the sample was determined by LC-MS/MS (described in Section 2.6) and NMR data compared with the literature data [24]. 

### 2.8. In Vitro Interleukin-6 (IL-6) mRNA Expression Assay 

The effects of LM and its derived compounds on *IL-6* mRNA expression in SCs were evaluated in vitro. Each sample was dissolved in water or DMSO and then resuspended in 20% FBS-Ham’s F-10 to the following final concentrations: LM (2.5 µg/mL), casuarinin (330 nM), hyperin, and quercitrin (220 nM each). The cells were prepared as described in Section 2.3. The supernatant of each culture was replaced with 20% FBS-Ham’s F-10 (Ctrl) or 20% FBS-Ham’s F-10 containing each sample described above and incubated for 16 h. Four independent cultures were performed for each treatment. The cultures were washed twice, and total RNAs were isolated using the RNeasy Plus Micro Kit (Qiagen, Hilden, Germany) according to the manufacturer’s protocol. RNA samples were quantified and qualified with a NanoDrop 2000 spectrophotometer (Thermo Scientific, Waltham, MA, USA), and then reverse transcribed using SuperScript IV VILO Master Mix (Invitrogen, Carlsbad, CA, USA) according to the manufacturer’s protocol. Quantitative real-time polymerase chain reaction (PCR) was performed using a QuantStudio 3 real-time PCR system (Applied Biosystems, Foster City, CA, USA). Reaction samples were prepared using TaqMan Fast Advanced Master Mix (Applied Biosystems) and TaqMan Gene Expression Assays (Applied Biosystems) according to the manufacturer’s protocol. The following two probes were used: glyceraldehyde-3-phosphate dehydrogenase (GAPDH) (Rn99999916_s1) and *IL-6* (Rn01410330_m1). *IL-6* gene expression levels were normalized to *GAPDH* using the ΔΔCt method. The data are shown as fold changes compared to the Ctrl.

### 2.9. In Vivo BrdU-Incorporation Assay 

The effects of LM and casuarinin on SC activation in rat skeletal muscle were evaluated in vivo by administration of each sample and BrdU to rats. The assay was performed according to a previously described method [19]. Twenty rats were divided into four groups (*n* = 5), and each group was orally administered water (Ctrl), LM (250 mg/kg/day, dissolved in water), or casuarinin (4 and 8 mg/kg/day, dissolved in water) daily for 4 d. Eight hours after the last administration, the animals were all intraperitoneally administered BrdU (50 mg/kg, dissolved in saline). The animals were sacrificed 16 h after BrdU administration, and SCs were isolated from the upper hindlimb muscle of each rat as described in Section 2.3. Cells suspended in 10% HS-DMEM were seeded into 48-well plates (three wells per rat) and cultured for 24 h. BrdU^+^ cells were detected by immunocytochemistry as described in Section 2.5, and the ratio of BrdU^+^ cells to the total number of cells was calculated. The values of the three wells for each rat were averaged and then further averaged for each group. The data are shown as fold changes compared to the Ctrl.

### 2.10. Statistical Analysis

Data are expressed as the mean ± standard deviation (SD). Statistical differences were analyzed by one-way ANOVA with Dunnett’s multiple comparison test and statistical significance was defined as *p* < 0.05. Standardized effect sizes (Hedges’ *g*) [25] were calculated and interpreted as small (0.20–0.49), medium (0.50–0.79), and large (≥0.80) according to the definition [26]. A correlation between casuarinin concentration and in vitro BrdU incorporation was analyzed using Pearson correlation. Statistical analyses were performed using the statistical software package R (version 4.0.2, https://www.R-project.org/, accessed on 27 February 2022, R Foundation for Statistical Computing, Vienna, Austria).

## 3. Results

### 3.1. LM Treatment Promotes the Proliferation of SCs but Not Myoblasts

**Figure 1:**
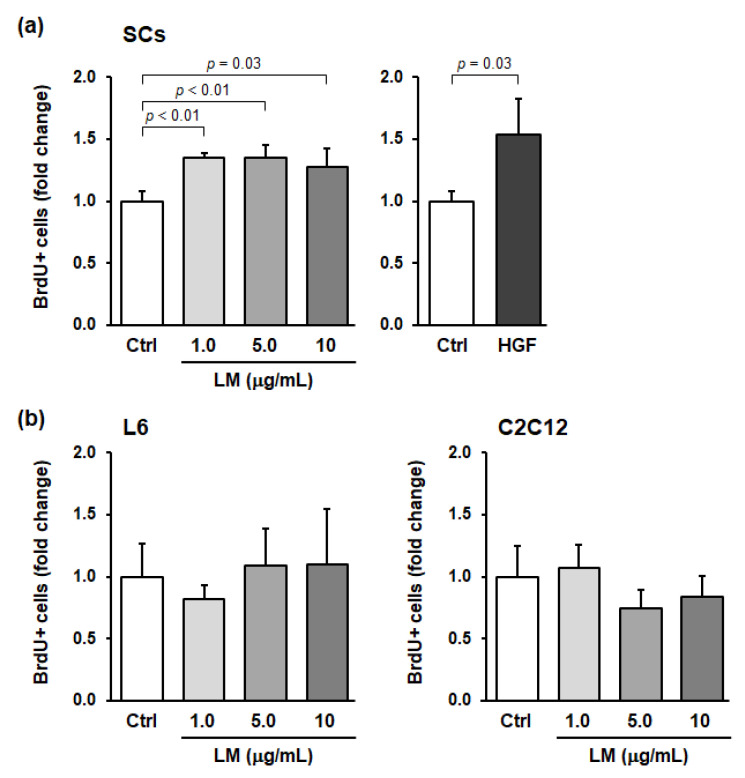
Lemon myrtle extract (LM) promotes BrdU incorporation into skeletal muscle satellite cells (SCs) but not in myoblasts in vitro. (**a**) Effects of LM and recombinant human hepatocyte growth factor (HGF) treatment on BrdU incorporation into SCs. (**b**) Effects of LM treatment on BrdU incorporation into L6 rat myoblast cells (left), and C2C12 mouse myoblast cells (right). SCs and myoblast cells were treated with LM or HGF for 22 h and then incubated with BrdU for an additional 2 h. The concentration of LM used is indicated in the figure. The HGF concentration used was 5 ng/mL. BrdU-positive (BrdU^+^) cells were detected by immunocytochemistry and the number of cells was counted. BrdU incorporation into each cell is expressed as BrdU^+^ cells per total cells and is shown as a fold change compared to the control (Ctrl). Data represent the mean ± standard deviation (SD) of three independent cultures. Significant differences (*p* < 0.05) relative to the Ctrl are indicated in the figure, and non-significant differences (*p* > 0.05) are not shown in the figure.

SCs are normally maintained in a quiescent and undifferentiated state, but when skeletal muscle is stimulated, SCs are activated and divide to produce myoblasts. We preliminarily evaluated the effects of approximately 200 plant extracts and phytochemicals on SC activation in vitro and found that LM activated SCs in vitro (data not shown). To investigate whether LM specifically activates SCs, we evaluated the effects of LM treatment on the activation of primary SCs and myoblast cell lines (L6 and C2C12) using in vitro BrdU-incorporation assays. Compared to the control, HGF treatment (positive control) significantly increased the ratio of BrdU^+^ SCs (*p* = 0.03, Hedges’ *g* = 2.07 (large)) (Figure 1a). LM treatment also significantly increased the ratio, with the effect plateauing at 1.0–10 µg/mL of LM (1.0 µg/mL: *p* < 0.01, *g* = 4.76 (large); 5.0 µg/mL: *p* < 0.01, *g* = 3.00 (large); and 10 µg/mL: *p* = 0.03, *g* = 1.85 (large)) (Figure 1a). In contrast, for myoblasts, LM treatment did not significantly change the ratio of BrdU^+^ cells at any concentration of LM (L6 and C2C12 myoblast cells: one-way ANOVA *p* = 0.67 and 0.24, respectively) (Figure 1b). Thus, we concluded that LM promoted cell cycle progression in SCs only.

### 3.2. Identification and Quantification of the Major Compounds in LM

**Figure 2:**
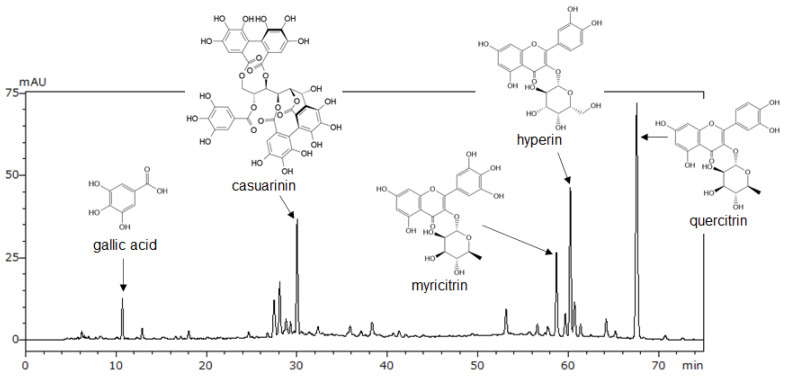
HPLC chromatogram of LM at 270 nm. Gallic acid, myricitrin, hyperin, and quercitrin were identified by comparison with standard compounds in HPLC analysis following LC-MS/MS. Casuarinin was identified by NMR analysis following LC-MS/MS.

We performed LC-MS/MS, HPLC, and NMR analyses to identify the major compounds in LM. First, five peaks were tentatively identified using LC-MS/MS (Supplementary Table S1). Next, using available analytical standards estimated from LC-MS/MS, we identified four of the peaks as gallic acid, myricitrin, hyperin, and quercitrin by HPLC analysis (Figure 2). However, an analytical standard of the fifth peak was not available; therefore, the peak was isolated from LM and analyzed using NMR. Based on ^1^H and ^13^C NMR spectra compared with literature data, we identified the fifth peak as casuarinin (Supplementary Table S2). Then, we determined the content of these compounds in LM using HPLC (Table 1). Among the five compounds, quercitrin had the highest content (38.8 mg/g) and gallic acid had the lowest content (2.5 mg/g) in LM. A representative HPLC chromatogram of LM with the identified peaks is shown in Figure 2.

### 3.3. Casuarinin Is the Active SC Growth Factor in LM

**Figure 3:**
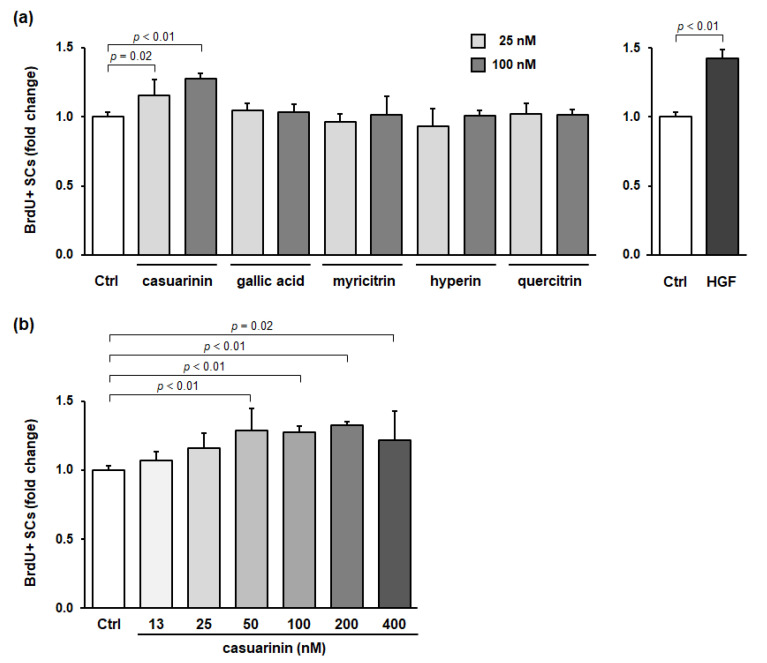
Casuarinin promotes BrdU incorporation into SCs in vitro. (**a**) Effects of the major compounds identified in LM on BrdU incorporation into SCs. (**b**) Effects of casuarinin concentration on BrdU incorporation into SCs. The concentration of each sample (except for HGF) is indicated in the figure. The HGF concentration used was 5 ng/mL. BrdU incorporation into SCs is expressed as BrdU^+^ SCs per total SCs and is shown as a fold change compared to the Ctrl. Data represent the mean ± SD of three independent cultures, except for Ctrl and HGF (nine independent cultures). Significant differences (*p* < 0.05) relative to the Ctrl are indicated in the figure, and non-significant differences (*p* > 0.05) are not shown in the figure.

To determine the active compound in LM involved in SC activation, we evaluated the effects of the major compounds identified in LM on in vitro SC activation. As well as HGF, casuarinin significantly promoted BrdU incorporation into SCs (*p* < 0.05), whereas the other four compounds did not (all *p* > 0.71) (Figure 3a). Casuarinin showed a significant increase in BrdU incorporation into SCs in the range of 50–400 nM (12.5 nM: *p* = 0.83; 25 nM: *p* = 0.12; 50 nM: *p* < 0.01, *g* = 3.31 (large); 100 nM: *p* < 0.01, *g* = 7.30 (large); 200 nM: *p* < 0.01, *g* = 9.56 (large); and 400 nM: *p* = 0.02, *g* = 2.03 (large)) (Figure 3b). There was little correlation between casuarinin concentration and BrdU incorporation (Pearson *r* = 0.22). The effective concentration of casuarinin (50–400 nM) corresponds to 47–375 ng/mL, calculated from its molecular weight of 936.7. From the effective concentration of LM (1–10 µg/mL, Figure 1b) and casuarinin content in LM (16.1 mg/g, Table 1), the effective concentration of casuarinin in LM was calculated to be 16–161 ng/mL, which was equivalent to the measured effective concentration of casuarinin described above (47–375 ng/mL, Figure 3b). Taken together, these data suggest that casuarinin is one of the active compounds in LM that is involved in SC activation in vitro.

### 3.4. Structurally Related Compounds of Casuarinin (Ellagitannins and Its Derivative) Fail to Promote SC Proliferation

Casuarinin is a *C*-glycosidic ellagitannin and consists of an open-chain form of glucose, two hexahydroxydiphenoyl (HHDP) groups, and a galloyl group. One HHDP group links to O-2 and O-3 of glucose (called the 2,3-HHDP group), the other HHDP group links to O-4 and O-6 of glucose (called the 4,6-HHDP group), and the galloyl group links to O-5 of glucose (called the 5-galloyl group). The 2,3-HHDP group is additionally linked to C-1 of glucose; therefore, casuarinin is called a *C*-glycosidic ellagitannin. 

**Figure 4:**
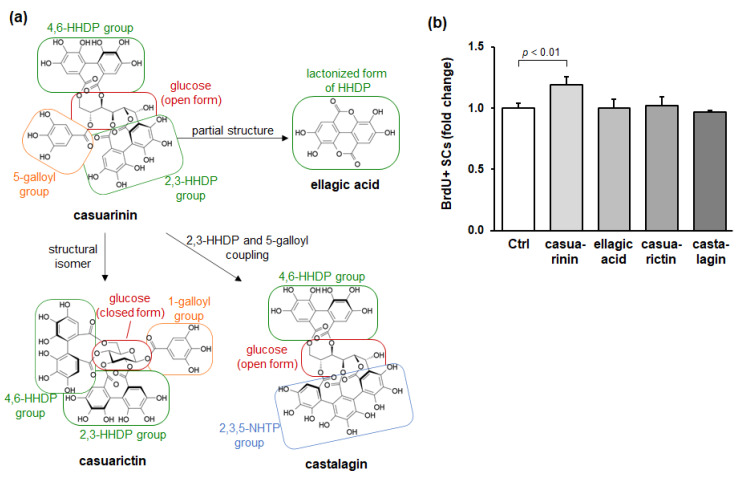
Structurally related compounds of casuarinin do not promote BrdU incorporation into SCs in vitro. (**a**) Chemical structures of the ellagitannins (casuarinin, casuarictin, and castalagin) and their derivative (ellagic acid). (**b**) Effects of the ellagitannins and their derivative (250 nM each) on BrdU incorporation into SCs. BrdU incorporation into SCs is expressed as BrdU^+^ SCs per total SCs and is shown as a fold change compared to the Ctrl. Data represent the mean ± SD of three independent cultures. Significant differences (*p* < 0.05) relative to the Ctrl are indicated in the figure, and non-significant differences (*p* > 0.05) are not shown in the figure.

To further investigate the effect of the casuarinin molecular structure on SC activation, we evaluated the effects of structurally related compounds of casuarinin on in vitro SC activation. Three compounds were chosen: (1) ellagic acid, a derivative of casuarinin; (2) casuarictin, a structural isomer of casuarinin; and (3) castalagin, an ellagitannin that has intramolecular coupling of the 2,3-HHDP and 5-galloyl groups in casuarinin. These structures are shown in Figure 4a. We found that casuarinin significantly promoted BrdU incorporation into SCs (*p* < 0.01), whereas the related compounds showed no effect (all *p* > 0.88) (Figure 4b). The structural similarities and differences between casuarinin and the related compounds are as follows: (1) ellagic acid is a lactonized compound of HHDP, which is a partial structure of casuarinin; (2) casuarictin has the same 2,3-HHDP and 4,6-HHDP groups as casuarinin, but differs from casuarinin in the number of hydroxyl groups and the position of the galloyl group due to the glucose moiety in closed form; and (3) castalagin has the same 4,6-HHDP group linked to the open-chain glucose moiety as casuarinin, but differs from casuarinin in the nonahydroxytriphenoyl (NHTP) group linked to the 2,3,5-positions of glucose moiety. None of these compounds activated SCs, suggesting that SC activation is not induced by all ellagitannins and their derivative, but rather by the casuarinin molecular structure—i.e., the 2,3-HHDP, 4,6-HHDP, and 5-galloyl groups linked to the open-chain glucose moiety.

### 3.5. LM or Casuarinin Treatment Upregulates IL-6 mRNA Expression in SCs

Skeletal muscle is an endocrine organ that produces and secretes various cytokines called myokines. Among the myokines, IL-6 is the first identified and the most studied myokine [27] and plays a key role in the regulation of muscle homeostasis and SC response [28]. Several studies have shown that IL-6 induces the activation and proliferation of SCs [29,30,31], and *IL-6* knockout experiments have shown that IL-6 is essential for SC activation and proliferation and subsequent muscle hypertrophy [30].

**Figure 5:**
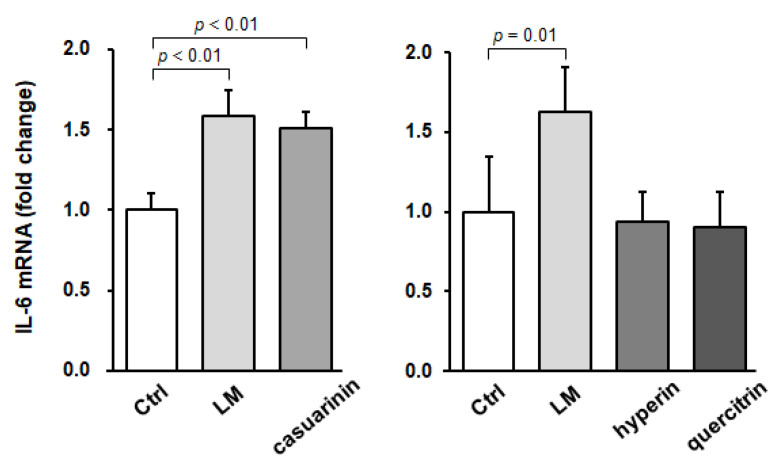
LM and casuarinin upregulate *IL-6* mRNA expression in SCs in vitro. SCs were treated with LM (2.5 µg/mL), casuarinin (330 nM), hyperin (220 nM), or quercitrin (220 nM) for 16 h and *IL-6* mRNA expression levels were quantified and normalized to *GAPDH*. Data are shown as fold changes versus Ctrl and represent the mean ± SD of four independent cultures. Significant differences (*p* < 0.05) relative to the Ctrl are indicated in the figure, and non-significant differences (*p* > 0.05) are not shown in the figure.

To investigate whether IL-6 is involved in SC activation by LM and casuarinin, we next evaluated *IL-6* mRNA expression in SCs in vitro. Isolated SCs were reseeded to enrich the cell density and incubated with each sample for 16 h, after which *IL-6* mRNA expression levels were quantified. Compared to the control, LM or casuarinin treatment significantly upregulated *IL-6* mRNA expression in SCs (LM: *p* < 0.01, *g* = 3.80 (large); and casuarinin: *p* < 0.01, *g* = 4.28 (large)) (Figure 5, left panel). In contrast, treatment with hyperin or quercitrin, which had no effect on BrdU incorporation into SCs (Figure 3a), did not affect *IL-6* mRNA expression (hyperin: *p* = 0.97, and quercitrin: *p* = 0.91) (Figure 5, right panel). Collectively, these data indicate that SC activation by LM and casuarinin is associated with upregulation of *IL-6* mRNA expression. 

### 3.6. Oral Administration of LM and Casuarinin Promotes SC Proliferation In Vivo 

**Figure 6:**
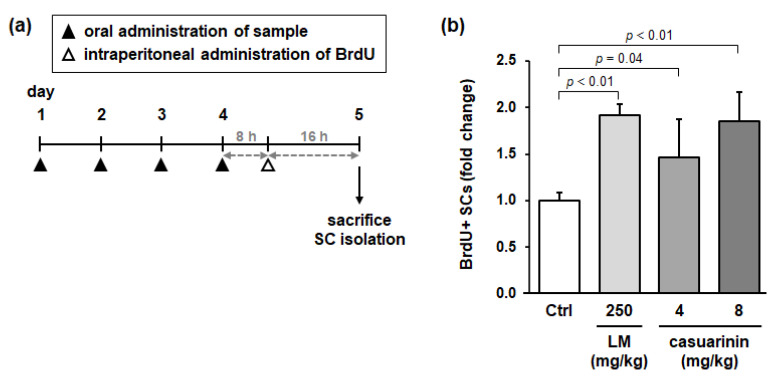
Both LM and casuarinin promote BrdU incorporation into SCs in vivo. (**a**) Experimental design of the BrdU-incorporation assay in rats. Each group of animals was orally administered water (Ctrl), LM, or casuarinin daily for 4 d. Eight hours after the last administration of each sample, all animals were intraperitoneally administered BrdU (50 mg/kg), and then 16 h later, animals were sacrificed and SCs were isolated. (**b**) Effects of LM and casuarinin administration on BrdU incorporation into SCs. The daily dose of each sample is shown in the figure. BrdU incorporation into SCs is expressed as BrdU^+^ SCs per total SCs and is shown as a fold change compared to the Ctrl. Data represent the mean ± SD of each group (three independent cultures per rat). Significant differences (*p* < 0.05) relative to the Ctrl are indicated in the figure, and non-significant differences (*p* > 0.05) are not shown in the figure.

To investigate whether LM and casuarinin activate SCs in vivo, we administered LM or casuarinin to rats and evaluated SC activation in rat skeletal muscle. The experimental design is illustrated in Figure 6a. Rats were divided into four groups (*n* = 5), and each group was orally administered water (as the control), LM (250 mg/kg/day), or casuarinin (4 and 8 mg/kg/day) daily for 4 d. The animals were then intraperitoneally administered BrdU and sacrificed 16 h later. Following SC isolation, the ratio of BrdU^+^ SCs was examined. Compared with the control group, animals administered LM or casuarinin (both doses) significantly increased BrdU incorporation into SCs (LM: *p* < 0.01, *g* = 7.80 (large); casuarinin (4 mg/kg): *p* = 0.04, *g* = 1.42 (large); and casuarinin (8 mg/kg): *p* < 0.01, *g* = 3.28 (large)) (Figure 6b). The casuarinin content in the effective dose of LM (250 mg/kg) was 4 mg/kg, which was consistent with the actual effective dose of casuarinin (4 or 8 mg/kg). Thus, we concluded that casuarinin is the active compound in LM that activates SCs both in vitro and in vivo.

## 4. Discussion

Lemon myrtle is a plant of the genus *Backhousia* in the family Myrtaceae, and its various solvent extracts, including a water extract, are known to have several biological activities, such as antimicrobial [32,33], anti-inflammatory [34,35,36], and antioxidant activities [35,36,37,38]. In the present study, we found that SC activation is a new biological activity of lemon myrtle water extract. Furthermore, we showed that lemon myrtle water extract did not activate myoblast cell lines in vitro. Contrary to our results, Sakulnarmrat et al. reported that an 80% aqueous methanol extract of lemon myrtle reduced the proliferation of colon, stomach, bladder, and liver cancer cell lines [39]. Although there are differences between the present study and the study by Sakulnarmrat et al. in evaluation methods—including solvents for extraction, cell culture, sample treatment, and measurement of cell proliferation—lemon myrtle extract may have a specific proliferative effect on SCs. Future studies are required to clarify the specificity of cell proliferation. 

Here, we identified five major compounds in LM: gallic acid, casuarinin, myricitrin, hyperin, and quercitrin. Among the five compounds, gallic acid, myricitrin, and hyperin have previously been identified in lemon myrtle [40,41,42]. To the best of our knowledge, casuarinin and quercitrin have been identified as constituents of lemon myrtle for the first time in this study. Furthermore, we investigated whether each major compound activated SCs or not. Based on three criteria (the effective concentration of LM, content of each compound in LM, and the evaluated concentration of each compound), we showed that only casuarinin activated SCs at the concentration contained in the effective concentration of LM. However, we cannot rule out the possibility that unidentified trace compounds present in LM may also activate SCs and that multiple compounds present in LM collectively regulate SC activation. Therefore, it is unclear whether casuarinin is the only active compound in LM. A comprehensive analysis of compounds present in LM and their activities will provide a deeper understanding of the active compounds present in LM. Interestingly, similar to LM, each of the five major compounds identified in LM has been reported to inhibit cancer cell proliferation [43,44,45,46,47,48,49,50]. In contrast, we showed that only casuarinin activated SCs, whereas the other four compounds did not. Therefore, the effects of LM and casuarinin on SCs may involve different mechanisms than their effects on other cells, including cancer cells. Evaluating the effects of each compound present in LM on various cells may help to clarify why LM is thought to activate only SCs. 

Casuarinin is a *C*-glycosidic ellagitannin that consists of an open-chain form of glucose, two HHDP groups, and a galloyl group. The molecular structure of casuarinin is quite different from that of the other four major compounds identified in LM, which do not activate SCs. Among the four major compounds, myricitrin, hyperin, and quercitrin are flavonol glycosides and differ from ellagitannins in terms of both their overall and partial structures. In contrast, gallic acid is a partial structure of ellagitannins but does not activate SCs. To examine the relationship between the molecular structure of casuarinin and SC activation, we evaluated whether structurally related compounds of casuarinin could activate SCs. First, ellagic acid, which is a lactonized compound of HHDP and is a partial structure of casuarinin, did not activate SCs. Since neither gallic acid nor ellagic acid induced SC activation, it is likely that the partial structure of casuarinin does not have SC activation properties. Next, casuarictin, which is a structural isomer of casuarinin, did not activate SCs. The differences in molecular structure between casuarinin and casuarictin are the glucose moiety (casuarinin has an open form, whereas casuarictin has a closed form), the number of hydroxyl groups (casuarinin has 16 OH groups, whereas casuarictin has 15), and the position of the galloyl group (in casuarinin, the galloyl group bonds to O-5 of glucose, whereas in casuarictin, it bonds to O-1 of glucose) (Figure 4a). Plaza et al. have shown that differences in the molecular structures of these isomers affect the strength of their antioxidant capacity [51]. Therefore, it is likely that the differences in the molecular structure between these isomers affect SC activation as well. Finally, castalagin, which is an ellagitannin with a 2,3,5-NHTP group, also did not affect SC activation. The 2,3,5-NHTP group in castalagin is the intramolecular coupling of the 2,3-HHDP and 5-galloyl groups in casuarinin. Casuarinin has a rigid 2,3-HHDP group and a rotatable 5-galloyl group, whereas castalagin has a rigid 2,3,5-NHTP group. Therefore, castalagin has a more rigid structure than casuarinin. Kaneshima et al. have shown that differences in the structural flexibility between casuarinin and castalagin may affect the strength of antioxidant activity [52]. Collectively, the molecular structure of casuarinin may contribute to the activation of SCs. It will be interesting to evaluate SC activation properties of analogs with partially substituted molecular structure of casuarinin.

IL-6 is the first identified and most studied myokine and plays a key role in the regulation of muscle homeostasis and SC response [27,28]. Several studies have shown the effects of IL-6 on SC and myoblast activation and proliferation. Kurosaka et al. reported that IL-6 treatment induced activation and proliferation of SCs in vitro [31]. Zhang et al. reported that in vitro myoblast activation by IL-6 is counteracted by an IL-6 neutralizing antibody [53]. Moreover, Weigert et al. showed that IL-6 treatment increased *IL-6* mRNA expression in myoblasts in vitro [54]. Collectively, these results suggest that the activation and proliferation of SCs and myoblasts by IL-6 are regulated in an autocrine manner via the upregulation of *IL-6* mRNA expression. Here, we investigated the involvement of IL-6 in SC activation by LM and casuarinin in vitro. We showed that LM or casuarinin treatment significantly upregulated *IL-6* mRNA expression in SCs, whereas hyperin or quercitrin treatment, which did not activate SCs, also did not change *IL-6* mRNA expression in SCs. These results suggest that the SC activation properties by LM and its major compounds are associated with *IL-6* mRNA expression in SCs. In contrast, animal and human studies have shown that IL-6 is involved in muscle hypertrophy mediated by SCs. Serrano et al. reported that compensatory hypertrophy increased *IL-6* mRNA and IL-6 protein expression in the muscle of normal mice and that *IL-6* knockout mice exhibited blunted muscle hypertrophy and suppressed SC activation and proliferation following compensatory hypertrophy [30]. McKay et al. demonstrated that muscle-lengthening contractions in humans increased the number of SCs, IL-6 protein levels in serum, and *IL-6* mRNA levels in muscle [55]. Begue et al. showed that acute resistance exercise in rats induces SC activation and increases *IL-6* mRNA levels in muscle and that 10 weeks of resistance exercise in rats induces muscle hypertrophy and a persistent increase in *IL-6* mRNA levels in muscle [56]. Taken together, these data suggest that IL-6 is involved in the mechanism of muscle hypertrophy via SC activation. Future studies are required to evaluate whether IL-6 is involved in the mechanism of SC activation by LM and casuarinin in vivo as well. 

Finally, we investigated whether LM and casuarinin activated SCs in vivo. Oral administration of LM (250 mg/kg) or casuarinin (4 and 8 mg/kg) to normal rats activated SCs. Similar to the in vitro data, the effective dose of casuarinin was consistent with the casuarinin content in the effective dose of LM. These results showed that casuarinin was the active compound in LM in vivo. To our knowledge, there are few functional food ingredients that activate SCs in the muscle of normal mice or rats. Oral administration of β-hydroxy-β-methylbutyrate (340 mg/kg) [57] or epigallocatechin-3-gallate (50 mg/kg) [58] to rats activated SCs in the muscle during recovery from atrophy but did not activate SCs in normal muscle. In addition, oral administration of proanthocyanidins (20 mg/kg) to rats [59] or intraperitoneal administration of epicatechin gallate (25 mg/kg) to mice [60] activated SCs in injured muscle, whereas intraperitoneal administration of resveratrol (20 mg/kg) or curcumin (1 mg/kg) to mice induced activation and proliferation of SCs in the atrophied muscle [61]. However, none of these ingredients have been reported to activate SCs in normal muscle. Although intraperitoneal administration of ursolic acid (200 mg/kg) to mice induced SC proliferation in normal muscle [62], it is unclear whether oral administration also activates SCs. Contrary to these studies, oral administration of LM or casuarinin to rats activated SCs in normal muscle. These results suggest that supplementation with LM or casuarinin in healthy adults may reduce the risk of developing sarcopenia and sarcopenia-related physical disability (e.g., frailty and sarcopenic obesity). In addition to the effects on normal muscle, supplementation with LM or casuarinin may improve sarcopenia and disuse atrophy by activating SCs in atrophied muscle and promote muscle regeneration by activating SCs in injured muscle. However, the fate of SCs activated by LM or casuarinin supplementation is unknown; that is, whether these activated SCs further proliferate and differentiate to become new myonuclei or proliferate but return to a quiescent state to maintain the SC pool. Future studies are required to assess the effects of LM or casuarinin supplementation on muscle hypertrophy. In addition, exercise induces muscle hypertrophy through both activation of SCs and stimulation of muscle protein synthesis; therefore, it is important to assess whether LM or casuarinin supplementation also stimulates muscle protein synthesis. Even if supplementation with LM or casuarinin do not affect muscle protein synthesis, supplementation with LM or casuarinin in combination with conventional nutrients that induce muscle protein synthesis, such as protein and leucine, may provide a powerful intervention for improving sarcopenia instead of exercise. 

## 5. Conclusions

In summary, we demonstrated that LM and casuarinin activated SCs both in vitro and in vivo. Our findings suggest that LM and casuarinin have potential as novel nutritional interventions for improving sarcopenia.

## Figures and Tables

**Table 1:** Content of the major compounds in LM.

Compound	Content (mg/g)
Gallic acid	2.5
Casuarinin	16.1
Myricitrin	12.6
Hyperin	19.1
Quercitrin	38.8

Data are shown as milligram of each compound per gram dry weight of LM.

## Data Availability

Data are contained within the manuscript and Supplementary Materials.

## Supplementary Materials

The following are available online at https://www.mdpi.com/article/10.3390/nu14051078/s1, Table S1: Tentative identification of major compounds in LM detected by LC-MS/MS; Table S2: ^1^H and ^13^C NMR spectra of casuarinin.
