# Supplementary material for: Lemon Myrtle (Backhousia citriodora) Extract and Its Active Compound, Casuarinin, Activate Skeletal Muscle Satellite Cells In Vitro and In Vivo
Source: Nutrients. 2022 Mar 4;14(5):1078. doi: 10.3390/nu14051078 (PMC8912364; doi:10.3390/nu14051078)
Supplement: Supplementary file 1 [file nutrients-14-01078-s001.zip › nutrients-1592745-supplementary.pdf]

Supplementary Materials

Table S1. Tentative identification of major compounds in LM detected by LC-MS/MS.

Compound	Retention time (min)	Molecular formula	[M−H]⁻ (*m*/*z*)	Fragment ions (*m*/*z*)
Gallic acid	9.6	$C_7H_6O_5$	169.0141	125.0236
Casuarinin	26.3	$C_{41}H_{28}O_{26}$	935.0769	300.9978<br>169.0133
Myricitrin	64.2	$C_{21}H_{20}O_{12}$	463.0862	316.0211
Hyperin	67.8	$C_{21}H_{20}O_{12}$	463.0862	300.0261
Quercitrin	84.5	$C_{21}H_{20}O_{11}$	447.0915	300.0262

Table S2. ^{1}H and ^{13}C NMR spectra of casuarinin (comparison of this study and reported data [24]).

^{1}H NMR position		δ (this study) (multiplicity, *J* [Hz])[a]		δ (reported) (multiplicity, *J* [Hz])[b]		Δδ[c]
2,3-HHDP	H-3′	6.39	(1H, s)	6.37	(1H, s)	0.02
4,6-HHDP	H-3	6.82	(1H, s)	6.81	(1H, s)	0.01
	H-3′	6.51	(1H, s)	6.49	(1H, s)	0.02
Galloyl	H-2,6	7.08	(2H, s)	7.07	(2H, s)	0.01
Glucose	H-1	5.52	(d, *J* = 5.0)	5.50	(d, *J* = 4.9)	0.02
	H-2	4.70	(dd, *J* = 2.1, 5.0)	4.68	(dd, *J* = 2.4, 4.9)	0.02
	H-3	5.38	(t, *J* = 2.0)	5.36	(m)	0.02
	H-4	5.45	(dd, *J* = 1.9, 8.7)	5.44	(dd, *J* = 1.2, 9.8)	0.01
	H-5	5.31	(dd, *J* = 3.0, 8.8)	5.30	(dd, *J* = 2.4, 8.5)	0.01
	H-6	4.91	(dd, *J* = 3.5, 13.4)	4.90	(dd, *J* = 3.7, 13.4)	0.01
	H-6	4.06	(d, *J* = 13.1)	4.04	(d, *J* = 13.4)	0.02

Table S2. (continued).

^{13}C NMR position		δ (this study)[a]		δ (reported)[b]		Δδ[c]
2,3-HHDP	C-1	117.0		117.0		0.0
	C-1′	116.7		116.7		0.0
	C-2	120.2		120.2		0.0
	C-2′	125.2		125.2		0.0
	C-3	118.0		118.0		0.0
	C-3′	105.3		105.3		0.0
	C-4	147.0		147.0		0.0
	C-4′	146.5		146.5		0.0
	C-5	140.1		140.1		0.0
	C-5′	135.9		135.8		0.1
	C-6	145.0		145.0		0.0
	C-6′	145.0		144.9		0.1
	C-7	167.1		167.0		0.1
	C-7′	171.0		171.0		0.0
4,6-HHDP	C-1	116.7		116.7		0.0
	C-1′	116.0		116.0		0.0
	C-2	127.6		127.6		0.0
	C-2′	127.1		127.1		0.0
	C-3	109.3		109.2		0.1
	C-3′	107.7		107.7		0.0
	C-4	146.0		145.9		0.1
	C-4′	146.0		146.0		0.0
	C-5	137.9		137.9		0.0
	C-5′	137.1		137.1		0.0
	C-6	144.4		144.4		0.0
	C-6′	144.5		144.5		0.0
	C-7	169.5		169.5		0.0
	C-7′	170.4		170.4		0.0
Galloyl	C-1	120.8		120.8		0.0
	C-2,6	110.5	(2C)	110.4	(2C)	0.1
	C-3,5	146.7	(2C)	146.7		0.0
	C-4	140.4		140.3		0.1
	C-7	167.2		167.2		0.0
Glucose	C-1	67.8		67.8		0.0
	C-2	78.1		78.0		0.1
	C-3	70.7		70.7		0.0
	C-4	74.8		74.8		0.0
	C-5	71.8		71.7		0.1
	C-6	65.2		65.1		0.1

Spectra were acquired in methanol-d4. [a] ^{1}H NMR (700 MHz) and ^{13}C NMR (176 MHz). [b] ^{1}H NMR (500 MHz) and ^{13}C NMR (125 MHz). [c] Difference between δ (this study) − δ (reported).
